# Scan Line Based Road Marking Extraction from Mobile LiDAR Point Clouds†

**DOI:** 10.3390/s16060903

**Published:** 2016-06-17

**Authors:** Li Yan, Hua Liu, Junxiang Tan, Zan Li, Hong Xie, Changjun Chen

**Affiliations:** School of Geodesy and Geomatics, Wuhan University, Wuhan 430079, China; lyan@sgg.whu.edu.cn (L.Y.); liuhua@whu.edu.cn (H.L.); tanjunxiang@whu.edu.cn (J.T.); lizan@whu.edu.cn (Z.L.); hxie@sgg.whu.edu.cn (H.X.)

**Keywords:** mobile LiDAR scanning, point clouds, scan line, road points extraction, road markings extraction

## Abstract

Mobile Mapping Technology (MMT) is one of the most important 3D spatial data acquisition technologies. The state-of-the-art mobile mapping systems, equipped with laser scanners and named Mobile LiDAR Scanning (MLS) systems, have been widely used in a variety of areas, especially in road mapping and road inventory. With the commercialization of Advanced Driving Assistance Systems (ADASs) and self-driving technology, there will be a great demand for lane-level detailed 3D maps, and MLS is the most promising technology to generate such lane-level detailed 3D maps. Road markings and road edges are necessary information in creating such lane-level detailed 3D maps. This paper proposes a scan line based method to extract road markings from mobile LiDAR point clouds in three steps: (1) preprocessing; (2) road points extraction; (3) road markings extraction and refinement. In preprocessing step, the isolated LiDAR points in the air are removed from the LiDAR point clouds and the point clouds are organized into scan lines. In the road points extraction step, seed road points are first extracted by Height Difference (HD) between trajectory data and road surface, then full road points are extracted from the point clouds by moving least squares line fitting. In the road markings extraction and refinement step, the intensity values of road points in a scan line are first smoothed by a dynamic window median filter to suppress intensity noises, then road markings are extracted by Edge Detection and Edge Constraint (EDEC) method, and the Fake Road Marking Points (FRMPs) are eliminated from the detected road markings by segment and dimensionality feature-based refinement. The performance of the proposed method is evaluated by three data samples and the experiment results indicate that road points are well extracted from MLS data and road markings are well extracted from road points by the applied method. A quantitative study shows that the proposed method achieves an average completeness, correctness, and *F*-measure of 0.96, 0.93, and 0.94, respectively. The time complexity analysis shows that the scan line based road markings extraction method proposed in this paper provides a promising alternative for offline road markings extraction from MLS data.

## 1. Introduction

Mobile Mapping Technology (MMT) emerged during the nineties in the twentieth century inspired by the availability of GPS technology for civilian uses. The world’s first mobile mapping system, named as GPSVan, which integrated a Code-Only GPS receiver, two CCD cameras, two video cameras and multiple dead reckoning sensors on a van, was integrated by Ohio State University in 1991 [1,2]. Later, The University of Calgary implemented the VISAT system including a dual-frequency carrier differential receiver and Inertial Measurement Unit (IMU) instead of the Code-Only GPS receiver and dead reckoning sensors, CCD cameras were also used in the VISAT system [3,4]. Similar systems were developed and reported in KiSS [5], GPSVision [6] and GI-EYE™ [7]. Since 2000, the main mapping sensors used in mobile mapping systems have been switched from cameras to laser range finders or laser scanners [8]. The mobile mapping systems equipped with mobile laser scanners are named Mobile LiDAR Scanning (MLS) systems, and most of the newly developed systems belong to this type.

MLS has been used in a variety of applications. The most obvious applications are road mapping and road inventory [9,10,11,12,13], in which road information such as curbstones, basic structures of road, road markings and traffic signs are extracted from MLS data. In [14,15], mobile LiDAR systems are used in tunnel cross-section and road cross-section measurements. In [16,17,18], the usage of mobile LiDAR systems in fluvial studies are reported. In [19], the railway infrastructure is automatically recognized from MLS data. In [20,21,22], mobile LiDAR systems are used for large scale plot mapping and individual tree crown reconstruction in forest research. In [23,24], MLS data are used to detect street environment changes and street-side vehicles. Geo-spatial information is extracted from MLS data for crowd control planning in [25].

Among the applications of MLS mentioned above, the most important and most promising applications are road mapping and road inventory, especially with the commercialization of Advanced Driving Assistance Systems (ADASs) and self-driving technology. In ADAS and self-driving, lane-level detailed 3D maps are needed for route planning and navigation in which the traditional 2D digital maps such as Google Maps are no longer sufficient. The most promising technology to generate such large scale lane-level detailed 3D maps is mobile LiDAR technology for its efficiency, cost-effectiveness, high accuracy and high density of the acquired point clouds.

To generate lane-level detailed 3D maps using mobile LiDAR point clouds, road markings and road edges should be extracted from the raw LiDAR point clouds. Road markings are generally made of high reflectivity material, so the intensity values of road marking points are theoretically higher than that of asphalt road surface points. Thus road markings can be distinguished from road points according to intensity information. However, the intensity values recorded by laser scanners are affected by target surface characteristics, acquisition geometry, instrumental effects and environmental effects [26]. In MLS, instrumental parameters, including the transmitted energy, the intensity bit depth and scaling, the amplifier for low reflective surfaces, the automation gain control, the brightness reducer for near distances and the aperture size, can be considered as constant, and the environmental parameters, including atmospheric transmittance and wetness, can be considered as constant in a scanning project, so the main parameters affecting the intensity values are target surface characteristics and acquisition geometry. As pointed out in [27], the incidence angle and scanning distance affect the intensity values of terrestrial laser scanning greatly. Thus the intensity values of road points vary not only because of different target characteristics (*i.e.*, asphalted road surface and road markings), but also because of different scanning incidence angle and scanning distance, making it difficult to separate road marking points from road points.

In order to extract road markings more efficiently and simplify the road marking points extraction algorithm, road points (including asphalted road surface points and road marking points) are first extracted from the raw LiDAR point clouds and then road markings are further extracted from the road points. In [28], road surface is segmented from the feature image generated by the raw 3D LiDAR points. In [29], road edges are extracted by combination of two modified versions of the parametric active contour model and road points can be extracted by the obtained road edges. In [30], road points are extracted by curbstone information on both sides of the road. In [15,31], road points are segmented from points in each scanner cycle by PCA analysis.

There are two typical types of methods to extract road markings from mobile LiDAR point clouds. The first type of methods convert the point clouds into georeferenced images and image processing methods are then used to extract road markings from the georeferenced images. In [11], 2D Georeferenced Feature (GRF) images are interpolated from the 3D road points by modified Inverse Distance Weight (IDW) interpolation, then Weighted Neighboring Difference Histogram is used to segment the GRF images and Multi-Scale Tensor Voting (MSTV) is used to extract road markings. In [30], geo-referenced intensity image is generated first by extended IDW method, and then road markings are recognized by a point-density-dependent multi-threshold segmentation of the intensity image and refined by morphological closing operations. In [31], road points are rasterized into images using a nearest neighbor algorithm to assign intensity data to a regular matrix, and then zebra crossings are detected by Standard Hough Transform from edges of the binary image. In [32], intensity curve fitting is used to reduce the variance of the intensity value, then intensity raster image is generated and road markings are extracted from the raster image by image processing method. In [33], candidate lane markings are localized in 3D by adaptive thresholding in scan lines, then 2D images are interpolated using the 3D candidate lane marking points and all candidate road markings are detected by Hough Transform clustering. Finally, false detections are eliminated by trajectory constraints and geometry checks. The second type of methods extract road markings from the 3D point clouds directly, which is much less reported until now. In [34], a method that extracts road marking points from 3D road points directly is reported. In this method, road points are divided into multi-segments, and road marking points are extracted from each segment by multi-thresholding method and refined by spatial density based filtering.

**Figure 1:**
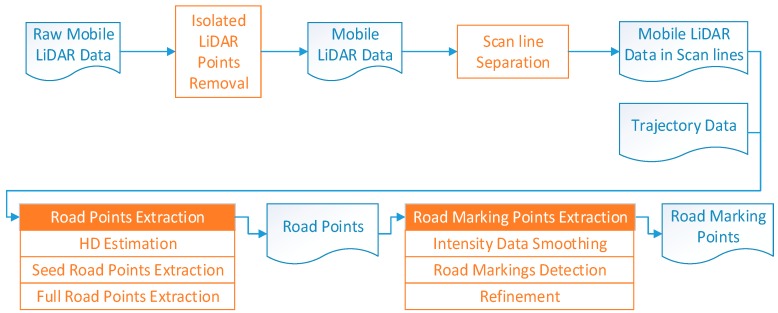
The workflow of the scan line-based road markings extraction method.

Extracting road markings by converting 3D point clouds into 2D georeferenced images simplifies the algorithms and many mature image processing algorithms can be used, but this method may lead to loss of high precision of the point clouds, and incompleteness and incorrectness of some objects. Extracting road marking points from 3D point clouds directly has seldom been reported. This paper proposes a scan line-based method to extract road marking points from 3D mobile LiDAR point clouds directly. The workflow of the method is illustrated in Figure 1. In this method, the acquired point clouds are first preprocessed to remove the isolated LiDAR points in the air and organized into scan lines, then the road points are extracted by moving least squares line fitting using the seed road points extracted by Height Difference (HD) between trajectory data and road surface. Finally, road marking points are extracted from scan line points by Edge Detection and Edge Constraint (EDEC) method after intensity smoothing and Fake Road Marking Points (FRMPs) are eliminated by segment and dimensionality feature based refinement.

This paper is structured as follows: Section 2 introduces our preprocessing methods including the method to remove isolated LiDAR points in the air from the raw LiDAR data and the approach to organize LiDAR point clouds into scan lines. In Section 3, the method to extract road points is addressed in detail and Section 4 focuses on the algorithms of road marking points extraction from road points and FRMPs elimination. Experiments, results and discussion are presented in Section 5. Finally, Conclusions are presented in Section 6.

## 2. Preprocessing

### 2.1. Isolated LiDAR Points Removal

**Figure 2:**
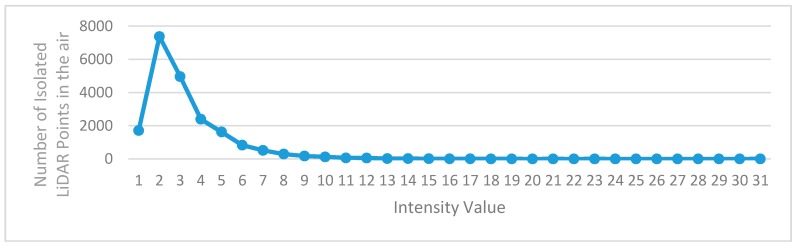
The intensity histogram of manually classified isolated LiDAR points in the air.

**Figure 3:**
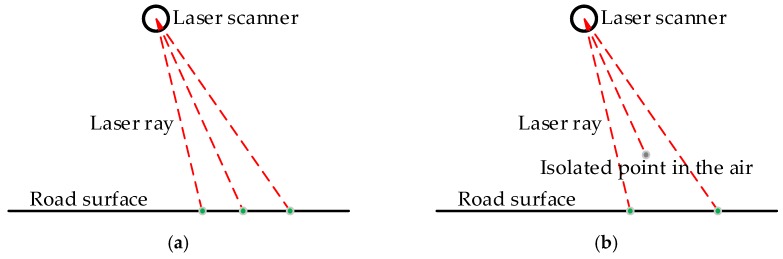
Three consecutive object points scanned by MLS: (**a**) three points are all road points; (**b**) one point is isolated point in the air with scanning distance obviously shorter than that of the previous point and the next point.

The isolated LiDAR points in the air occurring in the scanned point clouds affect the algorithms used to extract object information from point clouds. Actions should be taken to remove or reduce the isolated LiDAR points in the air before any subsequent operations. There are many causes for the isolated LiDAR points in the air, and the dust in the air is a case in point. No matter what the causes are, the intensity values of the isolated LiDAR points in the air are low. Figure 2 shows the intensity histogram of some isolated LiDAR points manually labeled from a dataset, which indicates that the intensity values of almost all isolated LiDAR points in the air are less than 10. This may be because that the isolated LiDAR points in the air are reflected by objects with very small diameter present in the air. Due to the small diameter, a very small part of the laser energy is reflected, leading to the observed small intensity values. Another characteristic of the isolated LiDAR points in the air is that their scanning distance (the distance from object to laser scanner) is obviously shorter than that of the previous scanned point and the next scanned point, as illustrated in Figure 3.

According to the two characteristics of the isolated LiDAR points in the air identified above, a point *p* with scanning distance $S_{c}$ and intensity value $I_{c}$, is classified as an isolated LiDAR point in the air based on Equation (1). In the equation, *S_p_* and *S_n_* correspond to the scanning distance of the previous point and the next point, and $\rho_{I}$ is the intensity threshold:

(1) $$\forall p:\{\begin{array}{ccc} if & \left(S_{c}<\;S_{p}\;\&\;S_{c}<\;S_{n}\;\&\;I_{c}<\rho_{I}\right) & \text{Isolated LiDAR point}\\ & otherwise & \text{Normal point} \end{array}$$

Some LiDAR points, especially tree points and vegetation points, may be mistakenly classified as isolated LiDAR points based on Equation (1), but this does not matter since we are only interested in road points and road points are seldom classified as isolated LiDAR points based on Equation (1).

### 2.2. Scan Line Separation

The original mobile LiDAR points are ordered sequentially by timestamp, and the point clouds can either be regarded as a whole or a large amount of points. In the principle of mobile LiDAR scanning, objects are scanned scan line by scan line with the movement of the platform. Scan lines can be recovered from the point clouds and subsequent processing can be done scan line by scan line. There are many advantages of processing mobile LiDAR points scan line by scan line: (1) it is easier to manage the small number of points in a scan line by simpler data structure than huge amount of points in a whole; (2) it is more time effective to process mobile LiDAR point clouds in scan lines compared with processing in a whole, especially with the increase of points number; (3) the scan lines can be processed in parallel.

**Figure 4:**
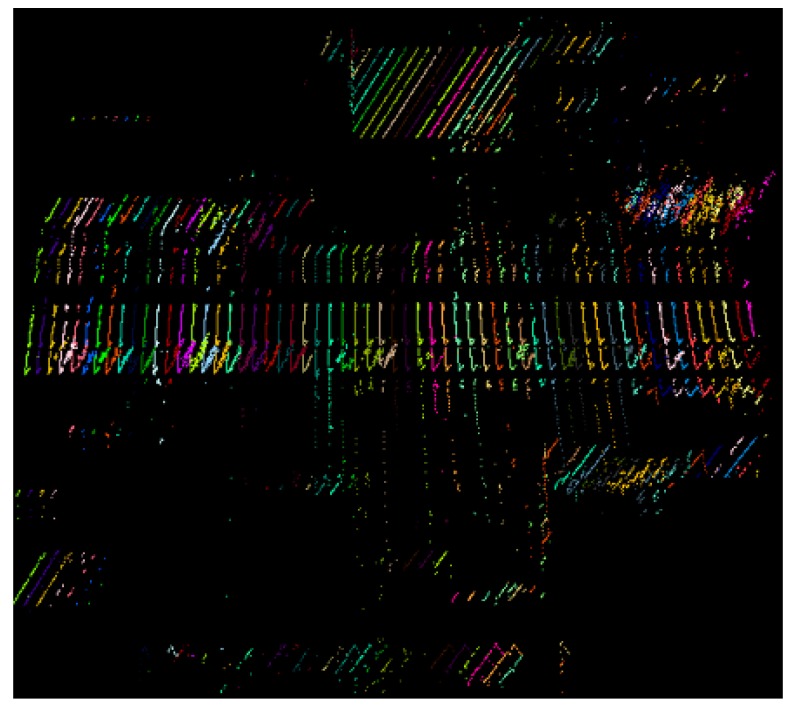
Point clouds colored by scan lines (one in every 50 scan lines is selected).

The scanning angle of a point *p* can be acquired from the raw LiDAR data or computed from its coordinates in Scanner Owned Coordinate System (SOCS). The scanning angles of points in each scan line increase consecutively from the minimum to the maximum in the Field Of View (FOV). For a mobile LiDAR with FOV of 360 degrees, the scanning angles of points in a scan line range from 0 to 360. The whole mobile LiDAR points can be separated into different scan lines according to the scanning angle. Figure 4 illustrates a small part of point clouds separated into scan lines. In order to show the scan lines more clearly, one scan line is selected in every 50 scan lines.

## 3. Road Points Extraction

**Figure 5:**
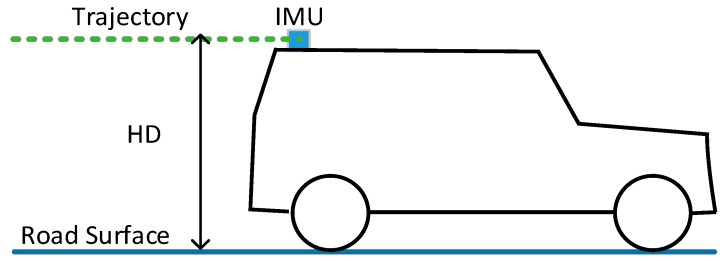
Height difference between trajectory and road surface.

The Position and Orientation System (POS) is rigidly fixed on the platform of a mobile LiDAR system. The points under the trajectory data are certainly road points. The Height Difference (HD) between trajectory and road surface (Figure 5) can be estimated based on the trajectory data and scanned mobile LiDAR point clouds. This HD can be used to help extract seed road points from scan line data effectively. After seed road points are extracted, full road points are extracted by moving least squares line fitting.

### 3.1. HD Estimation

Time data, position data(x, y, z) and attitude data (roll, pitch, heading) are included in every record of trajectory data; meanwhile, time data, coordinate data(x, y, z) and intensity data are included in every record of point clouds. In order to estimate HD, the scanned points right below the trajectory data should be found. Given a trajectory record with time $t_{0}$ and coordinate $\left(x_{0},y_{0},z_{0}\right)$, the points scanned within $t_{0}-\delta t$ and $t_{0}+\delta t$ can be extracted from the originally scanned point clouds, where *δt* is a small time value. Then the points horizontally neighboring the trajectory coordinate $\left(x_{0},y_{0},z_{0}\right)$ within a small distance $d_{0}$ are extracted. If *N* points are extracted, denote as $\left(x_{i},y_{i},z_{i}\right)$ and i∈1,2,3...N, HD can be estimated according to Equation (2):

(2) $$d_{hd}=\frac{\sum_{i=1}^{N}\left(z_{0}-z_{i}\right)}{N}$$

HD can be estimated for every scan line, but we usually estimate just one HD for each block of MLS data which is robust and more time efficient.

### 3.2. Seed Road Points Extraction from Scan Line

Seed road points can be extracted by HD. The trajectory data are outputted with constant time interval. Given a point *p* with time *t* and coordinate (x,y,z) in a scan line, there may be no trajectory record at exact time *t*. The trajectory data of time *t* should be interpolated from the trajectory records before *t* and after *t*. Assume that the interpolated trajectory coordinate is $\left(x_{t},y_{t},z_{t}\right)$, if the *z* value of *p* conforms to Equation (3), where *d_hd_* is HD estimated by Equation (2) and *ρ_hd_* is a small threshold, *p* is labeled as a seed road point:

(3) $$|z_{t}-z-d_{hd}|\;<\rho_{hd}$$

Some LiDAR points reflected by vegetation or other non-road objects may also have *z* values conforming to Equation (3) and are mistakenly labeled as seed road points. To identify the final seed road points, they are clustered into groups by their indexes. The points with consecutive indexes are divided into the same group, and the group with most point number is identified as the final seed road points.

### 3.3. Full Road Points Extraction from Scan Line

**Figure 6:**
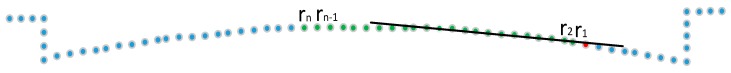
Fitting a line conform to the local part of the road surface. The green points are already identified road points and the red point is the point need to be determined whether it is a road point or not.

The whole road points in a scan line cannot be represented by a straight line, but a small part or a local part of the road surface can be fitted as a straight line. The distance from a point to the line fitted can be used to determine whether the point is road point or not, as shown in Figure 6. In order to identify whether the red point *p* in the right side of the extracted road points (colored in green) is a road point or not, a line can be fitted by the already identified road points near point *p*. If the distance from point *p* to the fitted line is smaller than threshold *ρ_d_*, the point is determined as road point, otherwise not. The line fitting will continue until two adjacent points are both determined as non-road points.

**Figure 7:**
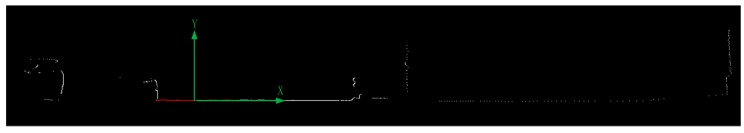
Scan line coordinate system to simplify line fitting.

In order to fit a line conforming to the local part of the road surface, moving least squares [35] line fitting is efficient. In moving least squares line fitting, the contribution of a point to the fitted line is determined by the weight. By assigning smaller weight to the points far away and greater weight to the points near the local part, the fitted line will conform to the local part. Besides, in order to simplify the line fitting, the points of the scan line are transformed to the 2D Scan Line Coordinate System (SLCS) whose origin is set at the middle of the seed road points and X axis parallel to the scan line direction, as illustrated in Figure 7. Equation (4) can be used as the line equation in SLCS.

If the already extracted road points are denoted as *r_i_*(*x_i_*, *y_i_*) in scan line coordinate system and the point nearest to *p* is *r*_1_, as illustrated in Figure 6. The line conforming to the local of the road at *p* can be fitted by minimizing Equation (5):

(4) l(x)=ax+b

(5) $$\sum_{i}w_{i}|l_{r_{1}}(x_{i})-y_{i}{|}^{2}$$

In Equation (5), *x_i_* and *y_i_* are the 2-D coordinate in SLCS and *w_i_* is the weight and calculated by the exponential weight function:

(6) $$w_{i}=e^{-\frac{{d_{i}}^{2}}{h^{2}}}$$

where *d_i_* is the distance between point *r_i_* and *r*_1_. In this way, the bigger the distance is, the smaller the weight is. Point *r*_1_, which is nearest to *p*, has a weight of 1 and other points have weights smaller than 1. *h* is the spacing parameter which can control within what distance the points has effect on the line to be fitted. Since *e*^−5^ equals to 0.0067, which is very small and almost have no effect on the fitted line, *h* can be determined by Equation (7) with the maximum distance *D* carrying effect:

(7) $$h=\frac{\sqrt{5}}{5}D$$

## 4. Road Markings Extraction and Refinement

### 4.1. Intensity Data Smoothing by Dynamic Window Median Filter

**Figure 8:**
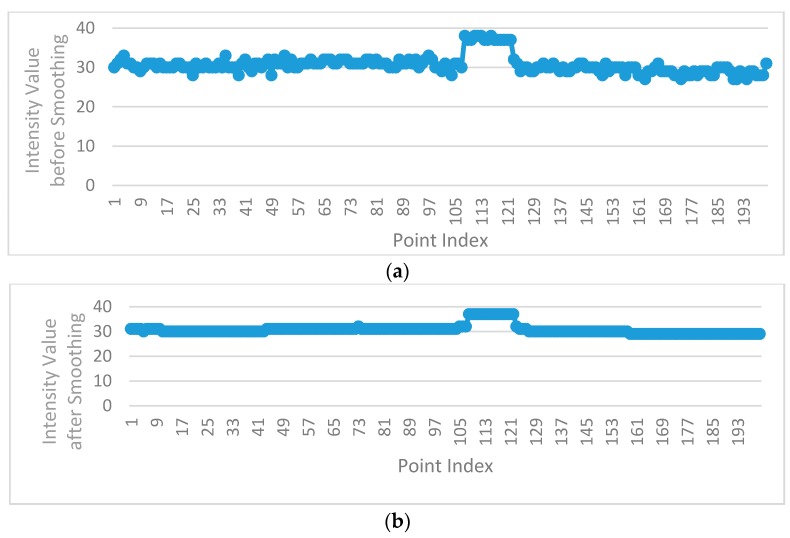
Intensity values of a scan line before and after dynamic window median filtering (**a**) Before filtering (**b**) After filtering by dynamic window median filter.

**Figure 9:**
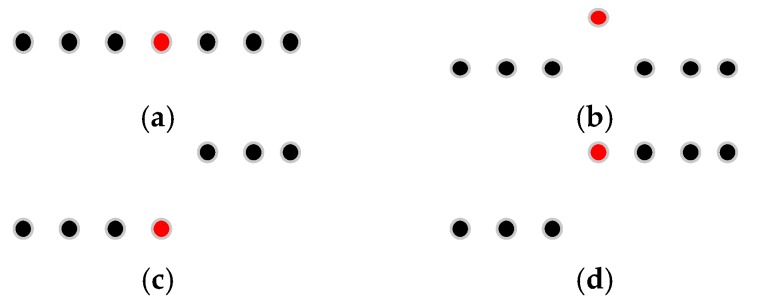
Median filter with window size of 7 and the points colored in red are points at the center of the window. (**a**) The intensity value of the center point is normal and will be kept; (**b**) The intensity value of the center point is obviously greater than other points and will be replaced; (**c**,**d**) The center point is at the edge of road marking and its intensity value will be kept.

The intensity values of road points in a scan line can be classified into two types: (1) the intensity values of road marking points; (2) the intensity values of asphalt road surface points. The intensity values of asphalt road surface points in a scan line (except the edges between road marking points and asphalt road points) should theoretically be the same or change smoothly, but in fact, the intensity values of adjacent asphalt road points in a scan line may vary greatly (Figure 8a). In order to smooth the intensity data of points in a scan line, median filter is used, which can not only smooth the intensity data of asphalt road surface points but also can preserve the intensity value of road marking edge point. Figure 9 shows four situations in filtering process by median filter with window size of 7. In Figure 9a,b, the points inside the window are all asphalt road points or all road marking points. In Figure 9a, the intensity value of the point at the center of the window is normal and will be kept. In Figure 9b, the intensity value of the point at the center of the window is obviously greater than that of others in the window and will be replaced by the median value of intensity values inside the window. In Figure 9c,d, the center point of the window is at the edge of road marking and the intensity value of the center point will be preserved after filtering since the window size is odd.

In order to preserve the intensity value of road marking edge points successfully, the window size should be smaller than the point number of road markings in the scan line. Because of different scanning distances, the distribution of mobile LiDAR points is non-uniform, and the point number of road markings varies in different scanning distances even when the width of the road markings are the same, so the window size of the median filter should be dynamically changed according to the point resolution. According to the standard for road markings [36], the minimum width of road markings in China is 0.2 m; thus in this paper, the window size of the median filter is dynamically determined by the minimum width and the point resolution. Figure 8b illustrates the intensity values after filtering by dynamic window median filter, with the intensity values of asphalt road surface points effectively smoothed and the intensity value of road marking edge point successfully preserved.

### 4.2. Road Markings Extraction by EDEC

**Figure 10:**
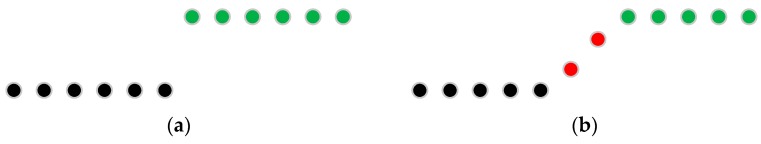
Steep edge and smooth edge between asphalt road points and road marking points: (**a**) Steep edge; (**b**) Smooth edge. The black points are low intensity points, the green points are high intensity points and the red points are transition points between high intensity points and low intensity points.

After smoothing by dynamic window median filter, the intensity values of points in a scan line are considered to change smoothly except at the edges between asphalt road surface and road markings. There are two types of edges between asphalt road surface points and road marking points as illustrated in Figure 10: (1) the steep edge; (2) the smooth edge. At the steep edge, the intensity value suddenly increases from low to high or otherwise decrease from high to low (Figure 10a). At the smooth edge, the intensity value changes smoothly from low to high or conversely from high to low (Figure 10b). Theoretically, all edges between road markings and asphalt road surface should be steep edges, but in fact, smooth edges also exist.

The road markings with steep edges can be extracted easily by thresholding, but those with smooth edges are hard to extract because the intensity values of road marking points are close to that of asphalt road surface points nearby. Road marking points are extracted from road points by detecting the edge points between road markings and asphalt road surface in a scan line in this paper. The edge points are detected by intensity gradient of the points in scan line. In order to detect smooth edge points successfully, the gradient value of *i*th point in scan line is estimated by:

(8) $$G_{i}=I_{i}-I_{i-k}$$

In Equation (8), *G_i_* is the gradient of *i*th point, and *I_i_* and *I_i−k_* are the intensity values of *i*th and (*i* − *k*)th point. Since the intensity values change smoothly after filtering, the absolute value of *G_i_* equals to zero or one at non-edge points and the absolute value of *G_i_* is greater than 1 at edge points. In order to detect edge points more conveniently, the gradients are further normalized to:

(9) $$N_{G_{i}}=\{\begin{array}{cc} 0 & \text{if}\;|G_{i}|\leq 1\\ G_{i} & \text{otherwise} \end{array}$$

**Figure 11:**
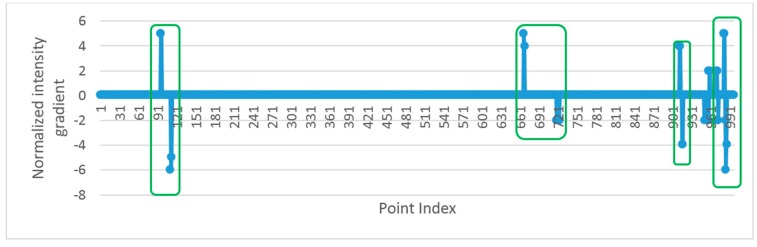
Normalized intensity gradient of road points in a scan line.

There are two edge points between a road marking and asphalt road surface, and the two edge points correspond to a pair of non-zero normalized gradient values with one positive and one negative. Figure 11 shows the normalized gradient values of a scan line, and the pairs of non-zero values inside the green rectangles represent the edge points of road markings. The pairs of non-zero values with one positive and one negative are detected from the normalized intensity gradient values, and the points between the two edge points are extracted as road marking points. This method is called Edge Detection and Edges Constraint method (EDEC). The extracted road marking points between a pair of edge points belong to a road marking segment.

### 4.3. Segment and Dimensionality Feature Based Refinement

Besides the road marking points, some asphalt road surface points are mistakenly extracted as road marking points by EDEC method. We call these mistakenly extracted points Fake Road Marking Points (FRMPs). The FRMPs are eliminated by segment based refinement and dimensionality feature based refinement. The segment based refinement is based on the fact that road markings are scanned in multiple consecutive scan lines while the FRMPs are scanned in a few consecutive scan lines or even only in one scan line. If a road marking extracted by EDEC is scanned by *M* consecutive scan lines and *M* is smaller than the minimum scan line threshold *ρ_s_*, the points consist of the road marking are classified as FRMPs and eliminated. *ρ_s_* is estimated by Equation (10) according to the minimum width of road markings along the platform moving direction (*W*_min_) and the distance between two scan lines (*D_r_*).

(10) $$\rho_{s}=\frac{W_{\min}}{D_{r}}$$

**Figure 12:**
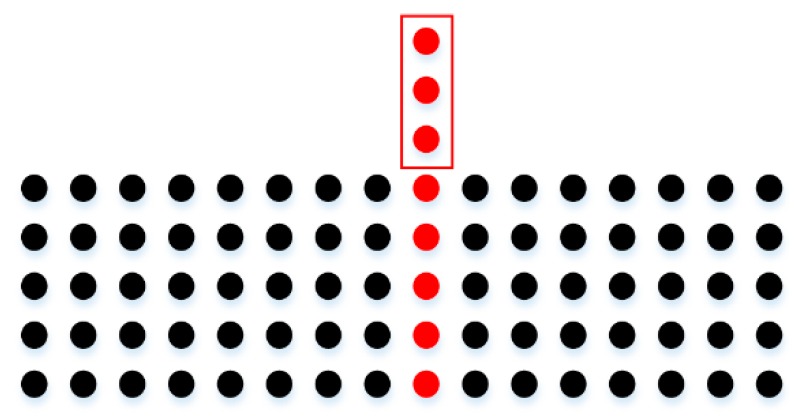
Road marking segment with FRMPs. The black points represent road marking segments without FRMPs. The red points represent road marking segment with FRMPs and the points inside the red rectangle are FRMPs.

Another type of FRMPs is illustrated in Figure 12. 

Such FRMPs can be eliminated by dimensionality feature [37] because the neighborhood of road marking points make up a 2D plane and the neighborhood of FRMPs make up a 1D line. The dimensionality feature linearity *L_λ_* is defined as follows:

(11) $$L_{\lambda }=\frac{\lambda_{1}-\lambda_{2}}{\lambda_{1}}$$

where $\lambda_{1}$, $\lambda_{2}$ and $\lambda_{3}$ are eigenvalues with $\lambda_{1}\geq \lambda_{2}\geq \lambda_{3}\geq 0$ estimated from the covariance matrix constructed for point p(x,y,z) by involving its neighborhood N(p). If $L_{\lambda }$ is greater than 0.9, point p is regarded as FRMPs and eliminated.

## 5. Results and Discussion

### 5.1. The Mobile LiDAR System and Mobile LiDAR Dataset

**Figure 13:**
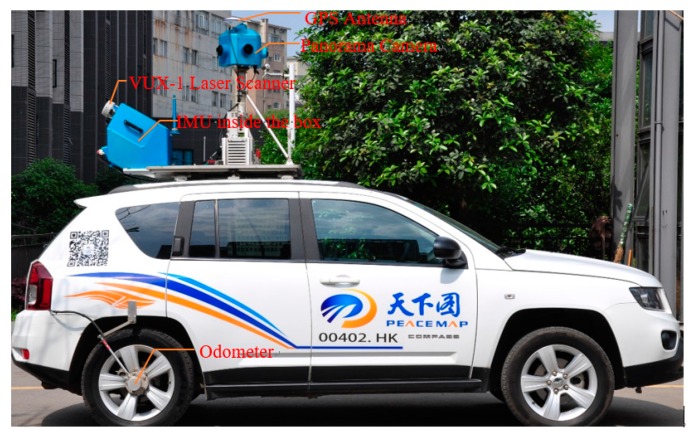
Mobile LiDAR system used for experiment dataset acquisition.

To evaluate the performance of the scan line based road markings extraction method proposed in this paper, a dataset is collected by a mobile LiDAR system operated by us (Figure 13). In this system, the SPAN-SE system with UIMU-LCI provided by Novatel Inc. in Canada, the VUX-1 laser scanner provided by RIEGL Laser Measurement Systems GmbH in Austria and a multi-camera panorama camera are integrated on a SUV car. In order to fuse the data in post-processing, all sensors are synchronized to GPS time. Besides, all sensors are configured through software run on the embedded computer and data sensed by the sensors are saved in the embedded computer. The boresight and misalignment between laser scanner and IMU are carefully calibrated before data collection.

The mobile LiDAR point clouds data of the Jincheng highway in Beijing (China) were collected by the mobile LiDAR system shown in Figure 13. The speed of mobile LiDAR system in data acquisition was about 40 kilometers per hour and the distance between two scan lines is about 0.06 m. The total data coverage is about 57 kilometers. To improve the accuracy and reliability of the mobile LiDAR point clouds, two GNSS base stations with data acquisition rate of 1 HZ were used during the data acquisition. The GNSS base stations were mounted along the highway and the baseline from mobile LiDAR system to the nearer GNSS base station was always shorter than 15 km. The GNSS data of base stations, the GNSS data and the IMU data of mobile LiDAR system were post-processed by Inertial Explorer software from Novatel to get optimized time, position and attitude data. The mobile point clouds were obtained by direct geo-referencing of the LiDAR sensed data using the optimized time, position and attitude data. Mobile point clouds scanned from both directions of the highway may suffer from inconsistency due to the error in position and attitude data [38,39,40]. The method in [39] was used to reduce the inconsistency of the point clouds scanned from both directions of the highway in our data.

**Figure 14:**
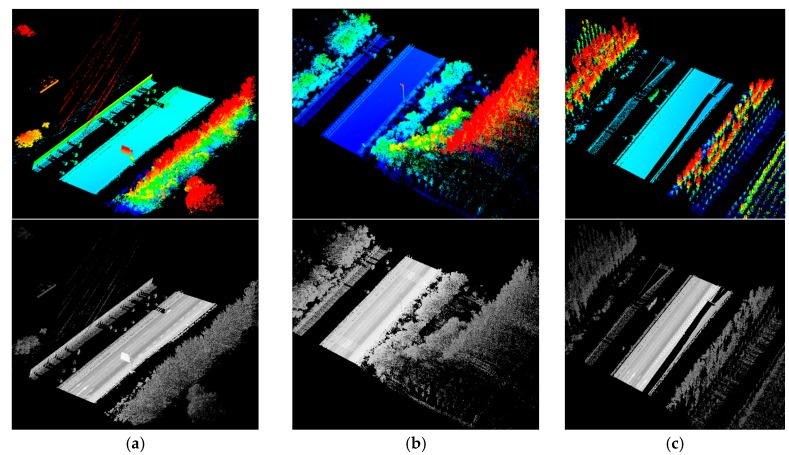
Three data samples selected from the dataset colored by elevation (first row) and intensity (second row): (**a**) data sample A; (**b**) data sample B; (**c**) data sample C.

For convenience, three typical data samples (data sample A, data sample B and data sample C as illustrated in Figure 14) are selected as test data from the whole dataset. Data sample A is about 100 m long and contains about 2.8 million points. Arrow shape and rectangle road markings are included in data sample A. Data sample B is about 70 m long and contains about 2.2 million points. Zebra crossings and other rectangle road markings are included in data sample B. Data sample C is about 100 m long and contains about 2.8 million points. Arrow shape road markings, rectangle road markings and exit signs are included in data sample C. 

**Figure 15:**
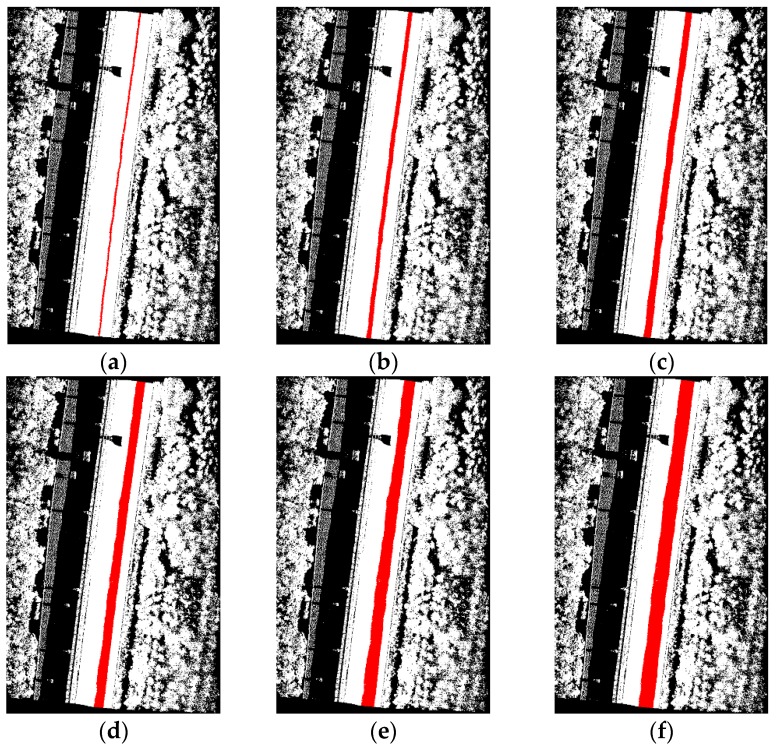
Seed road points extracted with different height difference threshold: (**a**) *ρ_hd_* = 0.01 m; (**b**) *ρ_hd_* = 0.02 m; (**c**) *ρ_hd_* = 0.03 m; (**d**) *ρ_hd_* = 0.04 m; (**e**) *ρ_hd_* = 0.05 m; (**f**) *ρ_hd_* = 0.06 m.

In the proposed road markings extraction method, the height difference threshold *ρ_hd_* in seed road points extraction, the distance threshold *ρ_d_* in full road points extraction and k in EDEC should be determined. In Section 5.2, a small block of mobile LiDAR dada is chose to analyze the parameter sensitivity and determine the empirical value of these parameters. The length of the chosen mobile LiDAR data is about 140 m and the number of points is about 4.8 million. Rectangle road markings and arrow shape road markings are included in the data. Besides, a car is scanned in the data and thus occlusion is existing in the chosen data (Figure 15). The chosen mobile LiDAR data for parameter sensitivity analysis is not included in the sample data used for quantitative evaluation in Section 5.3.

### 5.2. Parameter Sensitivity Analysis

The height difference threshold *ρ_hd_* will affect the point number of seed road points extracted. A smaller *ρ_hd_* will result in fewer seed road points and bigger *ρ_hd_* will result in more seed road points. We tested a set of parameter configurations on seed road points extraction and the testing results are shown in Figure 15. As seen from the seed road points extraction results, all configurations extract seed road points quite well, in which the bigger *ρ_hd_* is, the more seed road points are extracted. In order to ensure that enough seed road points are extracted and avoid non-road points being mistakenly extracted as seed road points, we set *ρ_hd_* to 0.03 m in our experiments.

**Figure 16:**
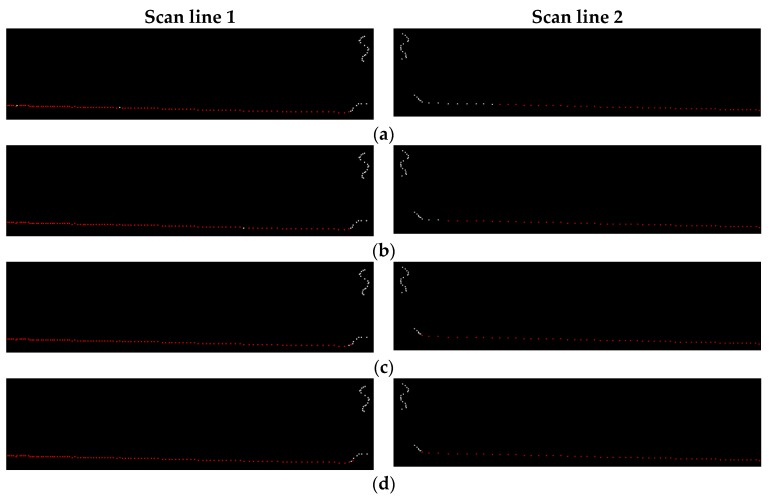
Road points (colored in red) extracted from two scan lines with different distance threshold: (**a**) *ρ_d_* = 0.01 m; (**b**) *ρ_d_* = 0.02 m; (**c**) *ρ_d_* = 0.03 m; (**d**) *ρ_d_* = 0.04 m.

The distance threshold *ρ_d_* in moving least squares line fitting for full road points extraction from scan lines will affect the road points extraction result. We tested a set of parameter configurations on full road points extraction to select an empirical *ρ_d_* value and the results of two scan lines are shown in Figure 16. From the results we can see that road points are not fully extracted when *ρ_d_* is set to 0.01 m and 0.02 m. When *ρ_d_* is up to 0.03 m, all road points are correctly extracted. When *ρ_d_* is increased to 0.04 m, no more points are extracted at these two scan lines. In order to ensure that all road points are extracted and avoid non-road points being mistakenly extracted as road points, *ρ_d_* is set to 0.03 m in our experiments.

**Figure 17:**
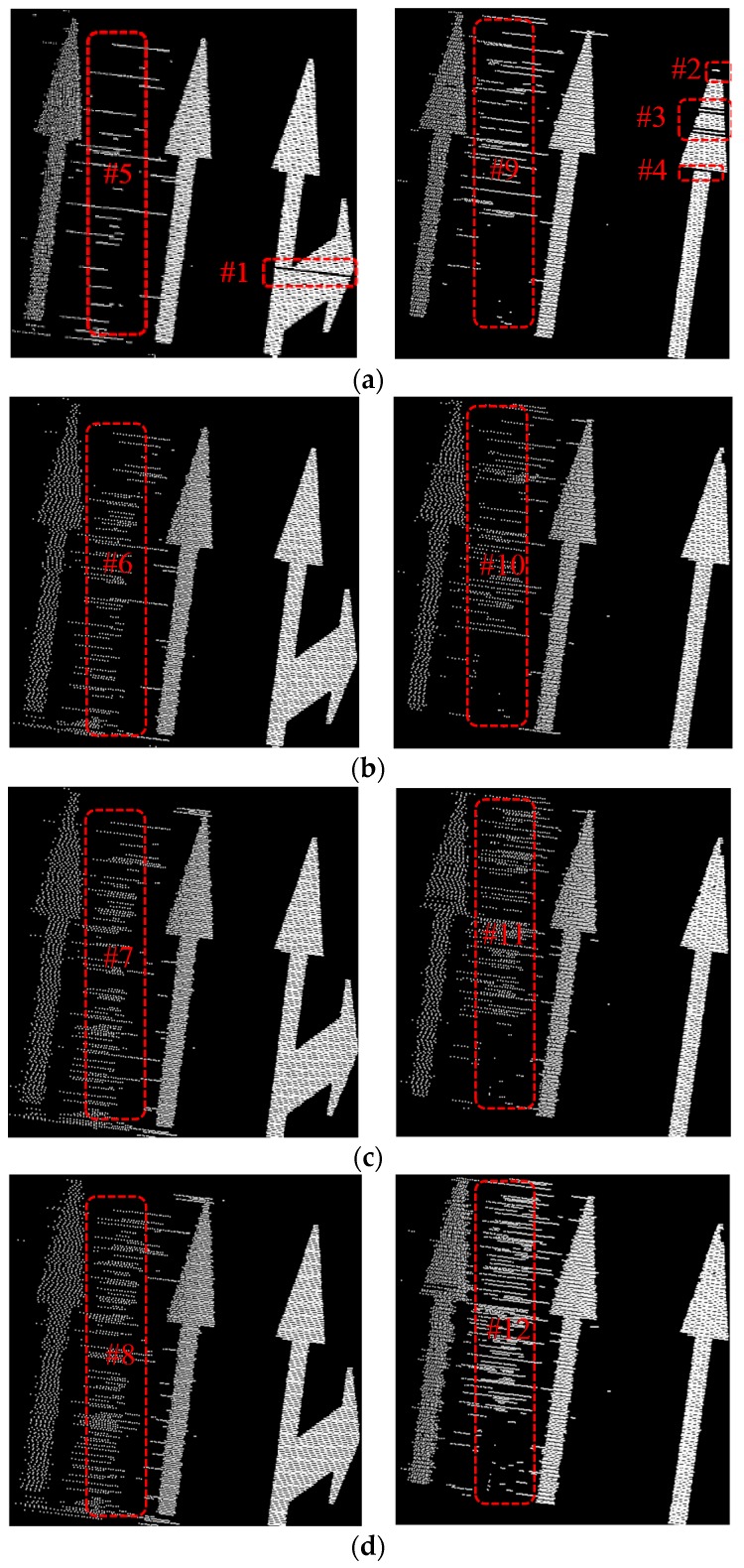
Road marking points extracted by EDEC with different *k* values (without segment and dimensionality feature based refinement): (**a**) *k* = 2; (**b**) *k* = 3; (**c**) *k* = 4; (**d**) *k* = 5.

Ideally, *k* with value 2 is enough to extract road marking points in EDEC, but some road marking segments may be missed because of the existence of smooth edges (some road marking segments are missed in #1, #2, #3 and #4 in Figure 17). With bigger *k* value, fewer road marking segments will be missed (none road marking segment is missed when *k* is set to 3, 4, and 5 in b–d), but more FRMPs appear in the result (it can be seen from #5, #6, #7, #8 and #9, #10, #11, #12 in Figure 17 where more FRMPs appear with the increase of *k* value). To extract road marking points more completely and to minimize the number of FRMPs, *k* is set to 3 in the experiments.

### 5.3. Road Points Extraction and Road Markings Extraction

The three data samples mentioned in Section 5.1 (data sample A, data sample B and data sample C) were used to evaluate the performance of the algorithms proposed in this paper. The parameters *ρ_hd_*, *ρ_d_*, *k* are analyzed in Section 5.2 and the configuration of these parameters in the experiments is based on the analysis. The minimum width of road markings along the platform moving direction is the width of stop line, which is 0.2 m in China [36], and the distance between two scan lines of the test data is about 0.06 meters, so *ρ_s_* is set to 3 in the experiments according to Equation (10). The parameter configuration of the experiments is illustrated in Table 1. 

**Figure 18:**
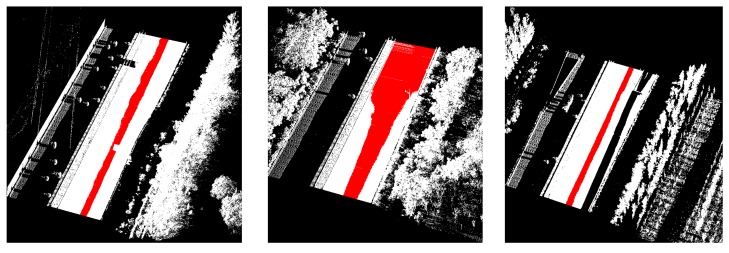

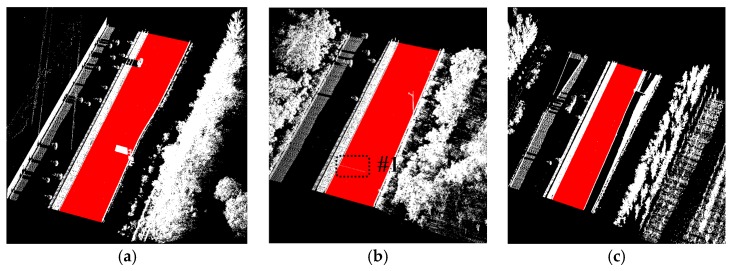
Seed road points (first row) and full road points (second row) extracted from data sample A (**a**); data sample B (**b**) and data sample C (**c**). The extracted seed road points and full road points are colored in red in the figure.

**Figure 19:**
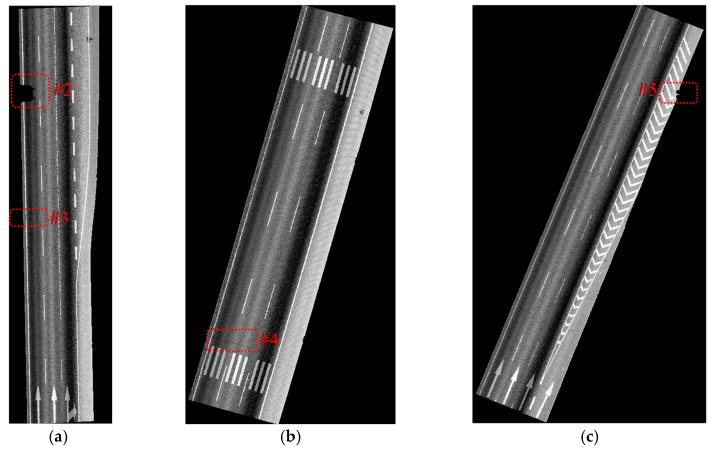
Full road points extracted by method proposed in this paper colored by intensity: (**a**) from data sample A; (**b**) from data sample B; (**c**) from data sample C.

**Figure 20:**
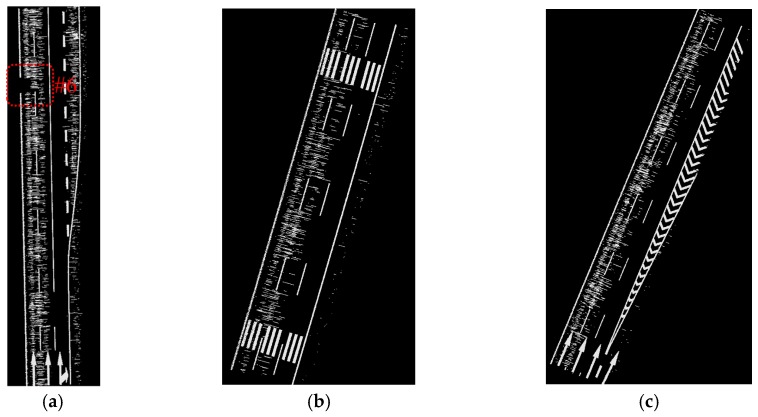
Road markings extracted by EDEC without segment and dimensionality feature based refinement: (**a**) from data sample A; (**b**) from data sample B; (**c**) from data sample C.

**Figure 21:**
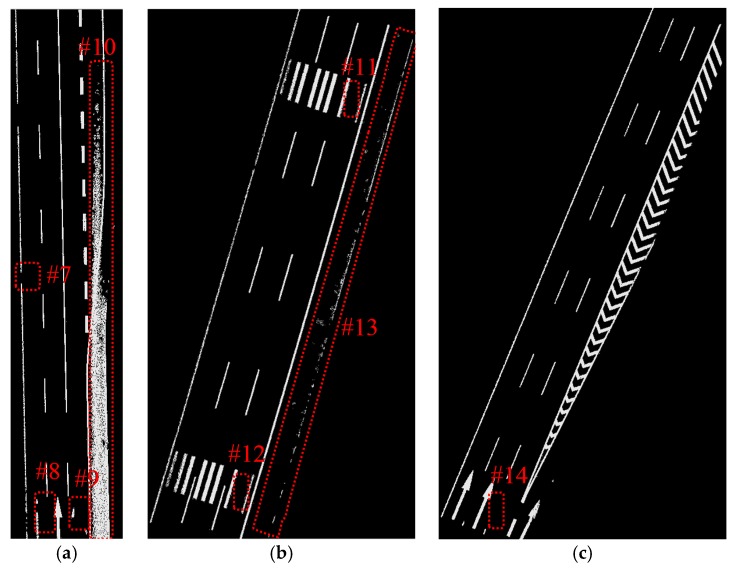
Road markings extracted by Yongtao’s method: (**a**) from data sample A; (**b**) from data sample B; (**c**) from data sample C.

**Figure 22:**
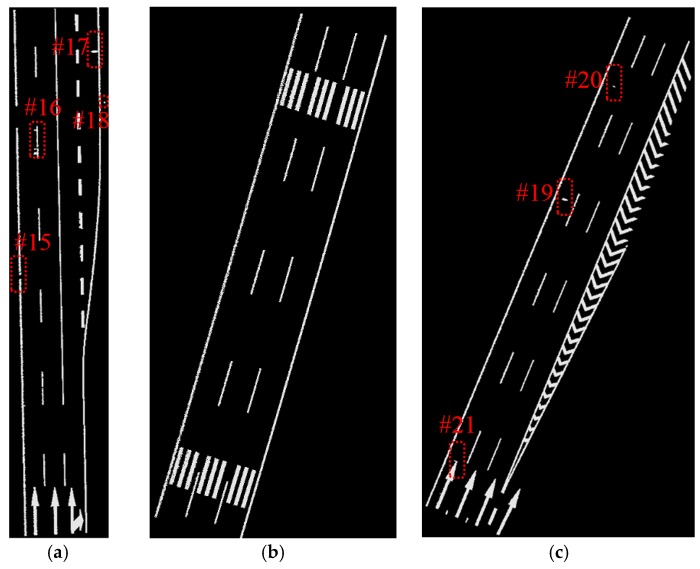
Road markings extracted by our method with FRMPs eliminated: (**a**) from data sample A; (**b**) from data sample B; (**c**) from data sample C.

**Figure 23:**
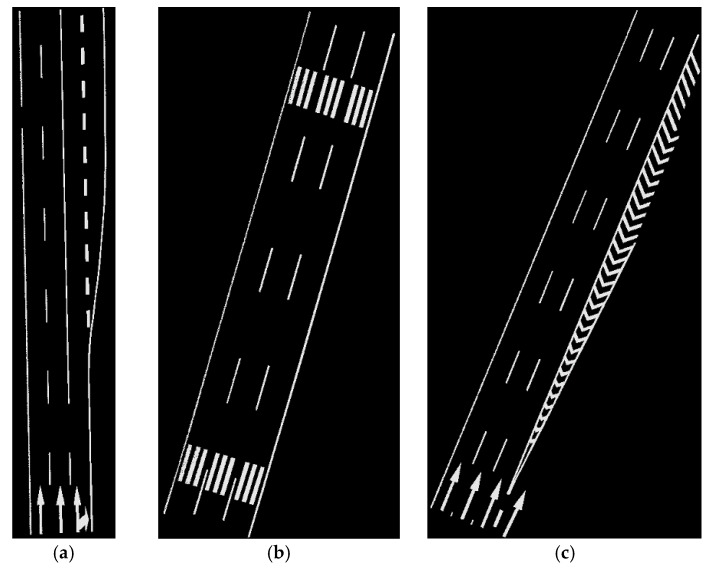
Road markings manually classified: (**a**) from data sample A; (**b**) from data sample B; (**c**) from data sample C.

The data samples were first preprocessed to remove the isolated LiDAR points in the air and organized into scan lines, then seed road points were extracted from the scan line by HD and full road points were extracted by moving least squares line fitting. The seed road points extraction and full road points extraction results are illustrated in Figure 18 and Figure 19. Finally, the intensity values were filtered by dynamic window median filter and road marking points were extracted by EDEC. FRMPs are eliminated by segment and dimensionality feature based refinement. The road marking points extracted by EDEC before and after segment and dimensionality based refinement are illustrated in Figure 20 and Figure 22 respectively. To compare our method with other methods, the road markings in the three data samples were also extracted using Yongtao’s method [34]. In Yongtao’s method, the road points were divided into 4 blocks. In [34], dataset was collected by a Riegl VMX 450 mobile LiDAR system. Since we use a different mobile LiDAR system and the scanning parameters can hardly be the same, the *ρ_SD_* in our experiments by Yongtao’s method was set to 11, which performs better in our dataset than the value recommended in [34]. The road markings extracted by Yongtao’s method and our method are illustrated in Figure 21 and Figure 22 respectively. Besides, the road markings in the three data samples were also manually classified from road points as reference data and illustrated in Figure 23. Quantitative evaluation of the extraction by our method and Yongtao’s method were conducted and the results are illustrated in Table 2. The computing time of our method for the three data samples are recorded to analyze the time complexity of our method and the results are listed in Table 3. As seen from the road points extraction results in Figure 18 and Figure 19, the seed road points are correctly extracted in every scan line in all three datasets, and the full road points are correctly extracted from three data samples except a part of one scan line missing in the road points extraction result of data sample B (#1 in Figure 18 and #4 in Figure 19). In Figure 19, there is no points in #2 and #5 because of moving car occlusion in data scanning and two cylindrical object placed on the edge of the road, respectively.

As seen from Figure 20, all road markings appeared in Figure 19 can be identified in Figure 20. Besides the road markings appear in Figure 19, many FRMPs are also mistakenly extracted as road marking points in Figure 20. There is no points in #6 in Figure 20 as a result of car occlusion in data acquisition.

Compared with the manually labeled reference data, the road markings extracted by Yongtao’s method miss road marking points in #7, #8, #9, #11, #12 and #14 in Figure 21 and many FRMPs exist in #10 and #13 in Figure 21. #7 and #9 are missed because the points are divided into blocks in Yongtao’s method and the intensity of these points are much lower than that of other road markings belong to the same block, especially #7 which corresponds to #3 in Figure 19. We can clearly identify that the intensity values of points in #3 in Figure 19 are lower than the nearby road markings. The right side of data sample A and data sample B are emergency lanes where vehicles are not allowed to run on except during an emergency. Similarly, vehicles are not allowed to run on exit lines on the right side of data sample C, so the road surface on the right side is less worn, leading to high intensity values of the points scanned on these areas. Due to the high intensity values of points scanned on the right side, many points are mistakenly extracted as road marking points (#10 and #13 in Figure 21) and some of the road markings belong to the same block are missed (#11, #12, #14 in Figure 21). Our method only misses a small area in #15 in Figure 22 which corresponds to #3 in Figure 19. Even after segment and dimensionality refinement, some FRMPs still exist in our result (#16, #17, #18, #19, #20 and #21 in Figure 22).

The method similar to [30] was used to evaluate the performance of the proposed algorithms quantitatively. Instead of rasterizing the extracted points into 2D georeferenced images directly, we first extract the actual road marking points from the extracted points manually. The actual road marking points and the manually classified reference data were rasterized into 2D georeferenced images using the rasterization method in [30]. The completeness (*cpt*), correctness (*crt*) and *F*-measure are defined as follows:

(12) $$cpt=\frac{C_{pixel}}{Rf_{pixel}}$$

(13) $$crt=\frac{C_{point}}{E_{point}}$$

(14) $$F=2\times \frac{\left(cpt\times crt\right)}{\left(cpt+crt\right)}$$

where *C_pixel_* and *Rf_pixel_* are the pixel numbers in rasterized georeferenced images of actual road markings extracted and manually classified reference data, *C_point_* and *E_point_* are the point numbers of actual road markings extracted and all road markings extracted. The results are illustrated in Table 2. It can be seen from Table 2 that the average *cpt*, *crt* and *F*-measure of Yongtao’s method are 0.85, 0.87 and 0.86 respectively and the average *cpt*, *crt* and *F*-measure of our method are 0.96, 0.93 and 0.94 respectively for these three data samples. The high *cpt* value of our method indicates that our method can extract road markings with little omission and the high *crt* value indicates that little FRMPs are remained in the extracted road marking points.

The computing time for road points extraction, road markings extraction and refinement are recorded to analyze the time complexity of our method. The proposed methods to extract road points and road marking points are implemented using C++ and running on an Intel(R) Core(TM) i7-3770 computer. Table 3 lists the computing time of the three data sample in seconds. It can be seen from the table that road points extraction is time consuming and the total time for each data sample is more than 40 seconds. The method proposed can be used to post-process mobile LiDAR point clouds which is not so critical for time complexity.

## 6. Conclusions

Extracting road markings from mobile LiDAR point clouds correctly and completely is still challenging. This paper proposed a scan line-based road markings extraction method from mobile LiDAR point clouds. The proposed method was applied to three data samples acquired by a mobile LiDAR system integrated by us. The experimental results indicated that the road points extraction method can effectively and accurately extract road points from the raw LiDAR point clouds, and the road markings extraction method can effectively extract road markings from the road points. The comparative study showed that the method proposed in this paper missed less road marking points, and less FRMPs are remained in the result. The quantitative study indicated that the method achieved average completeness, correctness, and *F*-measure of 0.96, 0.93, and 0.94 respectively, and outperformed Yongtao’s method.

## Figures and Tables

**Table 1:** Parameter configuration of the experiments.

*ρ_hd_*	*ρ_d_*	*k*	*ρ_s_*
0.03	0.03	3	3

**Table 2:** Quantitative evaluation results.

Data Sample	Data Sample A		Data Sample B		Data Sample C		Average	
Method	Yongtao’s	Our	Yongtao’s	Our	Yongtao’s	Our	Yongtao’s	Our
*cpt*	0.82	0.97	0.87	0.96	0.87	0.95	0.85	0.96
*crt*	0.70	0.92	0.95	0.93	0.96	0.93	0.87	0.93
*F*	0.76	0.94	0.91	0.94	0.91	0.94	0.86	0.94

**Table 3:** Computing time in seconds for road points extraction and road markings extraction.

Data Sample	Road Points Extraction	Road Markings Extraction and Refinement	Total
Data sample A	48.81	6.94	55.75
Data sample B	39.01	5.44	44.45
Data sample C	33.82	7.60	41.42
